# Qualitative Comparison of Image Stitching Algorithms for Multi-Camera Systems in Laparoscopy

**DOI:** 10.3390/jimaging8030052

**Published:** 2022-02-23

**Authors:** Sylvain Guy, Jean-Loup Haberbusch, Emmanuel Promayon, Stéphane Mancini, Sandrine Voros

**Affiliations:** 1 University Grenoble Alpes, CNRS, UMR 5525, VetAgro Sup, Grenoble INP, TIMC, 38000 Grenoble, France; sylvain.guy@univ-grenoble-alpes.fr (S.G.); jean-loup.haberbusch@univ-grenoble-alpes.fr (J.-L.H.); emmanuel.promayon@univ-grenoble-alpes.fr (E.P.); 2 University Grenoble Alpes, TIMA, 38031 Grenoble, France; stephane.mancini@univ-grenoble-alpes.fr; 3 University Grenoble Alpes, CNRS, UMR 5525, VetAgro Sup, Grenoble INP, INSERM, 38000 Grenoble, France

**Keywords:** laparoscopic surgery, distributed vision system, image stitching, panorama, simulated environment

## Abstract

Multi-camera systems were recently introduced into laparoscopy to increase the narrow field of view of the surgeon. The video streams are stitched together to create a panorama that is easier for the surgeon to comprehend. Multi-camera prototypes for laparoscopy use quite basic algorithms and have only been evaluated on simple laparoscopic scenarios. The more recent state-of-the-art algorithms, mainly designed for the smartphone industry, have not yet been evaluated in laparoscopic conditions. We developed a simulated environment to generate a dataset of multi-view images displaying a wide range of laparoscopic situations, which is adaptable to any multi-camera system. We evaluated classical and state-of-the-art image stitching techniques used in non-medical applications on this dataset, including one unsupervised deep learning approach. We show that classical techniques that use global homography fail to provide a clinically satisfactory rendering and that even the most recent techniques, despite providing high quality panorama images in non-medical situations, may suffer from poor alignment or severe distortions in simulated laparoscopic scenarios. We highlight the main advantages and flaws of each algorithm within a laparoscopic context, identify the main remaining challenges that are specific to laparoscopy, and propose methods to improve these approaches. We provide public access to the simulated environment and dataset.

## 1. Introduction

Laparoscopy is a widely used surgical technique that presents numerous advantages compared to laparotomy, especially in terms of recovery time and post operative pain [1]. Laparoscopy relies on an endoscope to visualise the operating field; therefore, its success is strongly related to the quality and extent of the intra-abdominal visualisation. Since the typical field of view (FoV) of an endoscope is quite limited, new devices have been developed to widen it. Some devices make use of dedicated optic lenses, such as panamorph lenses [2] or prisms [3], but often suffer from aberrations, distortions or the lack of miniaturisation. Robotic approaches using scanning fibres have also been developed [4,5], but they require complex and fragile elements that may impact their durability and cost.

### 1.1. Multi-Camera Systems

A recent approach developed to increase the FoV relies on the use of multi-camera systems, in which multiple miniature cameras are used conjointly to increase the single endoscope’s FoV. In [6], a modified trocar with two miniature cameras was evaluated through animal experiments. Preliminary works have shown a reduction in operating time, in the number of commands of a robotic endoscope holder [7], and a faster reaction to adverse events [8] in a testbench environment. In [9], a trocar–camera assembly deployed four miniaturised cameras, increasing the FoV of a 10 mm endoscope by 51%. In [10], a push-button was used to increase the space between two endoscopic cameras to widen the FoV. Most of these systems provide multiple video streams from the different cameras, but displaying these streams on multiple screens, as in prototype [6], is not optimal. It induces an increased mental workload for the surgeon, who needs to quickly apprehend how the scenes relate to each other. Therefore, it is crucial to be able to present a unique panorama for the surgeon before considering any concrete use of multi-camera systems in the operating room. This technique is called **image stitching**.

The challenge of **image stitching** is to build a panorama from a set of images taken from several viewpoints. The resulting panorama is made of outer areas, where camera images do not overlap, and a central area, where they do. Due to the inter-camera distances, an object may appear in different positions in the overlap area: this is called the parallax effect. When the inter-camera distance is small compared to the scene depth, parallax causes only a slight ghosting effect (blur). In laparoscopy, on the other hand, since the inter-camera distance is high and surgical instruments are very close, the parallax effect is much more significant. The challenge is to create a seamless image without parallax artefacts whilst retaining important information and keeping the main objects in realistic positions. A very similar technique called **image mosaicing** is already widely used in laparoscopy (e.g., [11,12,13,14,15]). Instead of stitching images from different cameras in the same time frame, it creates a panorama from the consecutive frames of a unique moving camera. Nonetheless, this technique fundamentally differs from our goal since the consecutive camera positions in mosaicing are relatively close to each other and there is almost no parallax, while in multi-camera systems the different viewpoints are far away from each other. Due to these differences, the methods developed to solve image stitching problems are different from those developed to solve mosaicing problems. Image mosaicing methods focus on the matching of a large number of images with overlaps but a small parallax and on homographies (e.g., [12]), while image stitching methods focus on the parallax problem.

### 1.2. Image Stitching in Current Multi-Camera Systems

There are few studies on multi-camera image stitching for laparoscopy. All methods roughly follow the same pipeline [16], using keypoint extraction (e.g., SIFT [17]) and matching, the alignment of images to a reference projection and blending to remove small misalignments. The alignment onto a reference projection is performed using one global transform called “homography”. We refer to this method as “global homography” later in this paper.

The prototype [9] followed the global homography pipeline with SURF [18] keypoint extraction, alignment with a homography and a final basic blending. This pipeline was only evaluated on simple testbench images and displayed strong discontinuities where some tool parts disappeared on the panorama.

The multi-camera system presented in [10] also used the global homography pipeline but improved it by using features extracted from disparity maps to obtain a more uniform keypoint distribution. A graph cut algorithm that was initially designed for texture merging [19] was also applied to remove ghosting. This algorithm finds the best plausible seam between two images, such that the stitching along that seam provides the most natural-looking result. This method successfully hid some parallax issues but could create discontinuities if images have not already been well aligned beforehand. The basic blending step of [9] was replaced with a more advanced multi-band blending [20]. Ref. [10] improved on the stitching result of the two previous multi-camera systems, but its prototype was not evaluated on realistic complex scenes that included visible tools.

### 1.3. Analysis

In the literature, no image stitching algorithm has been tested in realistic conditions, e.g., with organs and surgical instruments. The state-of-the-art stitching techniques for non-medical applications have also not yet been tested in laparoscopy. In parallel, publicly available datasets for the evaluation of these algorithms in laparoscopy are still missing. The closest publicly available dataset is the “Hamlyn Centre laparoscopic/endoscopic dataset”, which gathers publicly available stereoscopic recordings of laparoscopy. However, in a stereoscopic endoscope, the cameras are very close to each other in order to perform 3D reconstruction. These images exhibit almost no parallax, making this dataset unsuitable for the evaluation of image stitching in multi-camera prototypes. It is also not possible to simulate a multi-camera system by combining two images from this dataset that were taken at different time frames from the same camera. Indeed the scene, which is composed of deformable organs and moving surgical instruments, is not static.

### 1.4. Goals and Contributions

In light of this analysis, we defined two main goals highlighted in bold below. In Section 2, we review the main state-of-the-art techniques in the image stitching literature. In Section 3, we present **a simulated environment that generates realistic images, viewed from a multi-camera prototype, in a wide range of typical laparoscopic scenarios.** In Section 4 and Section 5, we **evaluate classical (i.e., currently used in laparoscopic multi-camera prototypes) and state-of-the-art image stitching methods from the non-medical literature** using our newly created dataset. Finally, we provide, in Section 6, some insights into the main limitations of current and state-of-the-art algorithms that are applied to laparoscopy. To our knowledge, this paper is the first to propose such an evaluation.

## 2. State-of-the-Art in Image Stitching

Image stitching for non-medical applications has been well studied the past decades, especially to build panorama images on smartphones. The global homography [16] method was one of the first approaches, but it was not designed to deal with parallax. Remaining small alignment issues are usually addressed using efficient blending algorithms, such as multi-band blending [20]. Graph cuts [19] are also used to address larger remaining alignment issues, although they may lead to visible discontinuities in the final panorama. From now on, we will use the term “Graphcut” (as a single word) to refer to this combination of global homography, graph cuts and blending, which is also the method used with prototype [10].

Recent approaches continue to follow the same pipeline consisting of the first step of image registration, followed by a graph cut or blending step. Their main improvement has been the replacement of the global homography registration with more complex space-varying models. These are divided into two categories: mesh and non-mesh based models. The first category relies on the continuous deformation of a mesh (grid) to smoothly align one image with another, while the second category directly computes the 2D deformation between images without using a mesh.

Among the mesh-based models, APAP [21] introduced the idea of dividing the image into a grid and computing local homographies for each quad cells of the grid. These local homographies have improved the alignment in the presence of parallax, but still display some projective distortions in non-overlapping areas. To lessen these distortions, many papers, such as PTIS [22], ANAP [23] and NIS [24], introduced a global similarity transform to guide the warping toward a more natural-looking result. Most of the recent approaches, such as PTIS [22], NIS [24] and REW [25], use the “Content-Preserving Warp” (CPW) [26] technique to improve their alignment quality. It consists of a pre-alignment step, followed by the smooth warping of the images using a specific energy function typically composed of: (a) an alignment term to match the keypoints between images; (b) a local similarity term to reduce local distortions; and (c) a global similarity term to reduce the global distortions on the image. CPW has achieved good alignment performance, with few distortions in the presence of parallax. Among these mesh-based methods, PTIS [22] stands out by being a seam-driven stitching technique. Instead of considering the graph cut algorithm as an independent post-processing operation, this algorithm searches for the optimal alignment such that graph cut could work optimally.

Apart from these mesh-based techniques, some non-mesh-based models directly compute 2D deformations on the whole overlap of the images. In [27], later referred to as the Perazzi method, optical flow was computed in the overlapping area to warp and align the images. This deformation was then smoothly extrapolated into non-overlap areas to avoid discontinuities.

Finally, very recent methods use deep learning approaches to solve the image stitching problem. Some works [28,29,30] only focused on the homography estimation problem using supervised and unsupervised deep learning methods, while others [31,32,33] later developed supervised neural architectures for the whole image stitching pipeline. However, supervised approaches are not optimal since they are trained using synthetic ground truth datasets. These datasets do not offer a complete equivalent for multi-camera scenarios as they do not reconstruct a reference panorama, but rather they are generated using an artificial camera point of view that encompasses the whole equivalent panorama FoV, which is not perfectly representative of the parallax error. This is later referred to as a “non-parallax” view. A very recent article proposed an unsupervised deep image stitching framework [34], later referred to as the UDIS method, for image stitching that does not require a supervised synthetic dataset. The UDIS framework is composed of: (a) an initial neural network for the global homography estimation and (b) a second architecture for alignment refinement with reconstruction networks. Out of all deep learning approaches, we focus on this unsupervised approach since it is the most recent and promising method.

The main characteristics of these algorithms are summarised in Table 1.

Concerning the metrics, none are commonly accepted in the literature. Many papers based their evaluations on qualitative analyses. Some papers developed their own quantitative metrics, but these were specific to each algorithm or limited to keypoint-based algorithms.

This review shows that many papers in the non-medical image stitching literature have improved the methods currently used in laparoscopy. To evaluate how these new methods behave in our domain, we propose to build a realistic dataset of images from multi-camera systems in laparoscopic situations. This is the contribution of the next section.

## 3. Material: A New Simulated Environment

The development of image stitching techniques in laparoscopy is slowed down for two reasons. Firstly, there is no public dataset of images recorded from multi-camera systems that could be used to evaluate the image stitching algorithms. Secondly, it is difficult and time-consuming to record new in vivo images for each new configuration of a multi-camera system to check the quality of the image stitching. This is why we developed a flexible simulated environment that offers realistic laparoscopic images and is easily configurable to take many laparoscopic scenarios into account.

**Figure 1:**
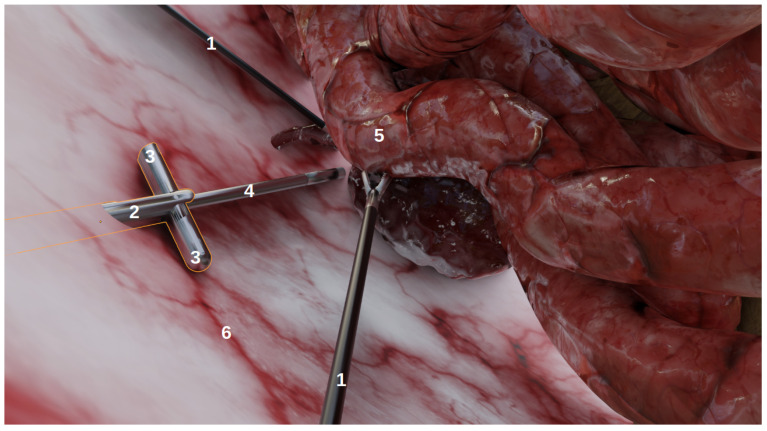
The simulated environment viewed from a user perspective in Blender. This is not a camera perspective and thus, does not include the rendering (e.g., illumination, shadows). (1) The laparoscopic forceps, (2) the multi-camera prototype, (3) two deployed cameras, (4) the endoscope, (5) organs, (6) the abdomen.

The goal of this environment, developed using Blender (http://www.blender.org (accessed on 6 January 2022)), a 3D modelling and rendering software, was not to achieve anatomical realism, but rather to produce images that could realistically represent laparoscopic conditions as viewed from laparoscopic cameras. As shown on Figure 1, it contains:

- Organs, with corresponding realistic image textures, e.g., intestines, blood vessels, abdomen;
- Tools: laparoscopic forceps and a 5 mm endoscope with LEDs;
- Multi-camera prototypes, where the number of cameras and their focals, FoVs and inter-spaces can be modified. In this study, we simulated the multi-camera prototype of [6] with two deployable Misumi TD-VBL31105L-77 cameras (1.83 mm focal, 69° × 43° FoV) and an inter-space of 4.5 cm. These specifications were established according to the results of a specifically performed experiment, provided in Supplementary Material Figures S4 and S5, which showed that this was a good compromise between overlap and the enlargement of the field of view.

Along with the environment, we developed a software to easily design new scenes, where the following parameters could be specified:

- Tools: e.g., the number/position/orientation of forceps;
- Endoscope: the depth inside the abdomen cavity, camera parameters (e.g., focal, resolution), the power of the LEDs;
- Multi-camera system: the position/orientation of the device, camera parameters (e.g., focal, resolution), the power of the LEDs;
- Rendering parameters: the type of rendering engine, output image resolution and exposure.

**Figure 2:**
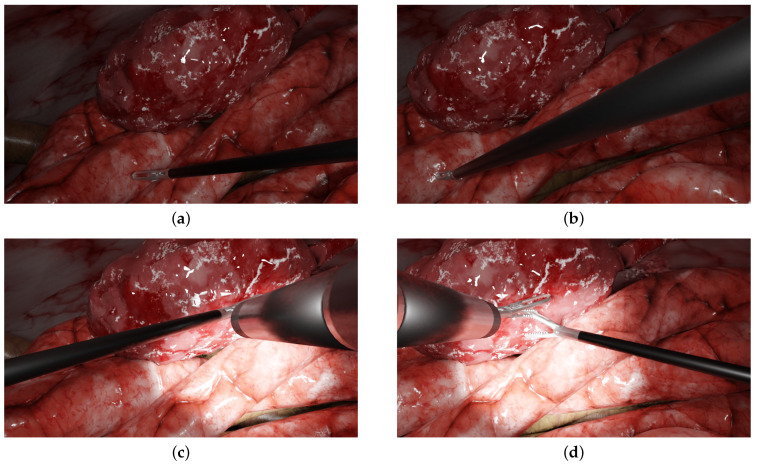
Examples of typical laparoscopic situations: images from a multi-camera system rendered in the simulated environment. The left and right columns display the left and right simulated images of the prototype, respectively. (**a**,**b**) One forceps with no endoscope (**c**,**d**), two forceps, one endoscope and a halo.

Some examples of the generated images are shown in Figure 2 and other examples are provided in Supplementary Material Figure S1. Videos can also be generated, with moving forceps or endoscopes, for instance. The simulated environment and some videos/images are publicly available at the following link: https://gricad-gitlab.univ-grenoble-alpes.fr/guys/laparo_simulated_environment (accessed on 6 January 2022). Another main benefit of using this environment is that early versions of multi-camera prototypes can be evaluated within realistic laparoscopic contexts (various scenarios, tools configurations, etc.) and can be simulated accordingly without requiring the manufacturing of new prototypes and the realisation of new in vivo experiments.

In the next section, we present our evaluation of several image stitching algorithms using this realistic dataset with various levels of parallax and scene complexity.

## 4. Benchmarking of Image Stitching Algorithms

### 4.1. Methodology

Our objective was to evaluate: (a) the performance of image stitching algorithms that are currently used with multi-camera prototypes for laparoscopy and (b) the performance of recent non-medical image stitching algorithms within the laparoscopic context in order to test whether they could improve on those currently used in laparoscopy. Based on the analysis shown in Table 1, we list the expected problems and their impacts within the laparoscopic context in Table 2: background blur (often on background organs), distortions (projective or non-projective), duplicated elements, disappearing elements and discontinuities. In the following, we define a “perfect stitching” as a stitching that shows none of the problems listed in Table 2 and a “perfect alignment” as a stitching that shows only projective or non-projective distortions. The most important problems within a laparoscopic context are those highlighted in the red box in Table 2, i.e., duplicated or disappearing elements, such as tools, since they can be a source of significant confusion for the surgeon. An incorrect rendering of the instruments, for instance, is unacceptable for clinicians as this would make their handling more challenging and impact their perception of depth.

Due to the lack of a standard quantitative metric, as explained in Section 2, the evaluation of the different algorithms was performed in relation to these problems. We implemented a module on the CamiTK platform [35] that could test multiple image stitching algorithms:

- Global homography [16];
- Graphcut (global homography and graph cut [19]);
- Mesh-based models: APAP [21], ANAP [23], NIS [24], REW [25] and PTIS [22];
- Two non-mesh-based models: the optical flow-based model Perazzi [27] and the neural network-based model UDIS [34].

These algorithms were selected because they cover the actual algorithms used with prototypes and the main recent advances in image stitching for non-medical applications. Out of all the deep learning approaches detailed in Section 2, we chose to evaluate the unsupervised approach UDIS [34] since it does not require a synthetic ground truth dataset (which are “no parallax” biased) and seems more promising for future application in laparoscopy.

Out of all these methods, PTIS [22] and Perazzi [27] were implemented since their authors did not provide any source codes. For clarity purposes, algorithms that have already been used with laparoscopic prototypes before are dash-underlined for the rest of the paper, i.e., global homography used in prototype [9] and Graphcut used in prototype [10].

All algorithms, except for UDIS that does not include an explicit blending step, were evaluated with a multi-band blending post-processing, since that has been established as the optimal blending algorithm in terms of quality/speed trade-off [36]. Noticing that the best results among the mesh-based models were given by REW [25], only the REW results are shown in this section. The results of other mesh-based methods (APAP [21], ANAP [23], NIS [24] and PTIS [22]) are provided in Supplementary Material Figures S2 (temple scenario) and S3 (laparoscopic scenario).

### 4.2. Experiments

**Figure 3:**
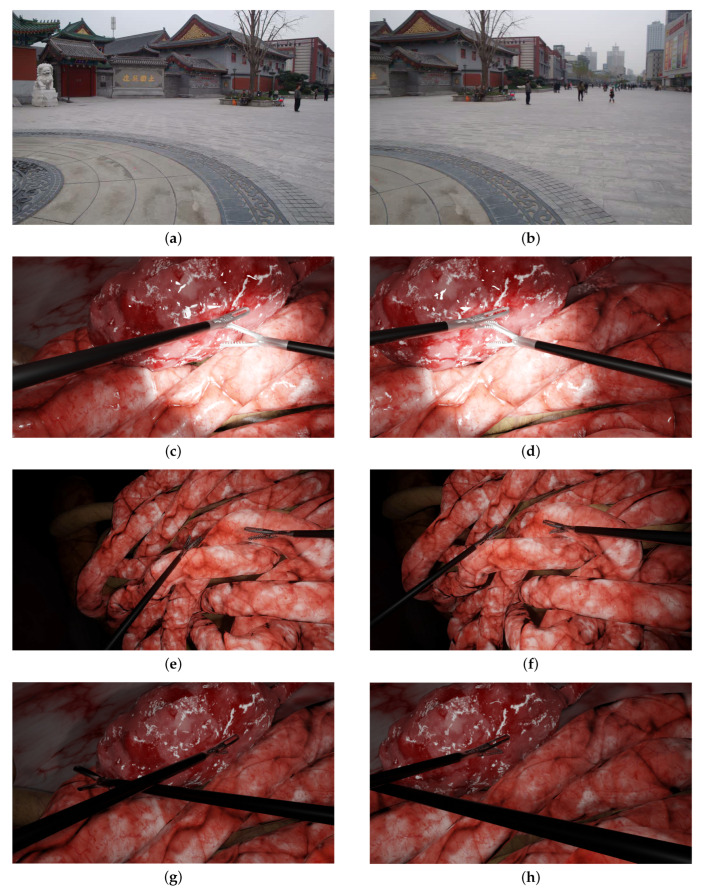
The scenarios used in the experiments. The left and right columns show the left and right images, respectively: (**a**,**b**) typical non-laparoscopic situation of a temple; (**c**,**d**) two tools with medium parallax; (**e**,**f**) two tools with high parallax; (**g**,**h**) two intersecting instruments.

A first experiment (Section 5.1) evaluated the algorithms in a non-laparoscopic situation to assess their performance within the context for which they were designed. This temple scenario is a classic example used in non-laparoscopic image stitching [37] (cf. Figure 3a,b). In a second experiment (Section 5.2), these methods were evaluated in laparoscopic scenarios, from simple scenes to more complex scenes: Figure 3c,d two tools with medium parallax; Figure 3e,f two tools with high parallax (more depth of field and relief in background); and Figure 3g,h two intersecting instruments.

## 5. Results

### 5.1. Experiment 1: On a Non-Laparoscopic Scenario

**Figure 4:**
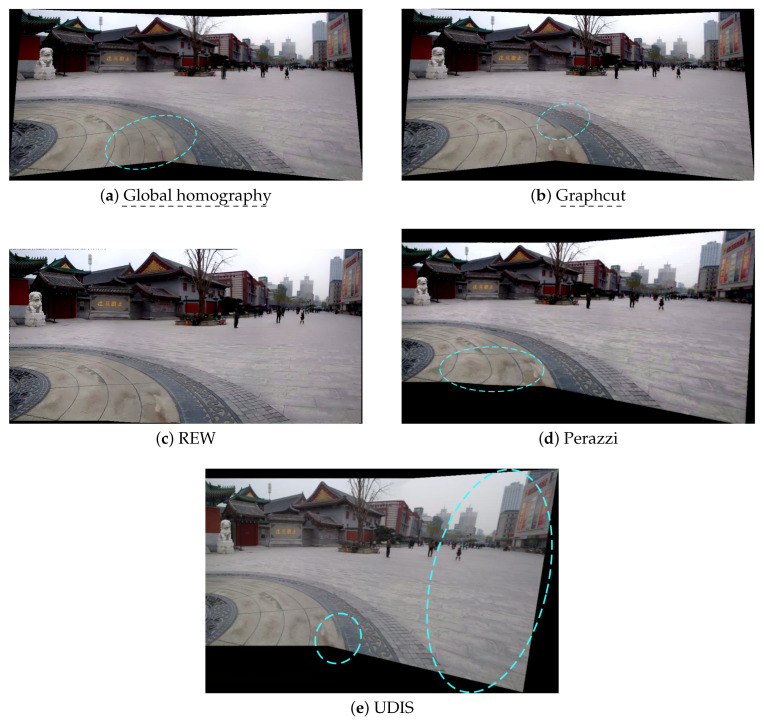
The resulting panoramas in the temple scenario. Blue dashed circles point to the main issues. (**a**) Global homography: background blur, duplicated elements, projective distortions. (**b**) Graphcut: no background blur, but strong discontinuities and some projective distortions. (**c**) REW: perfect stitching. (**d**) Perazzi: perfect alignment, but slight distortions. (**e**) UDIS: no background blur, but a few discontinuities and some projective distortions.

Before evaluating these methods in laparoscopic situations, we benchmarked them in situations for which they were designed, i.e., in outdoor photography, such as the temple scenario in Figure 3a,b. The results are displayed in Figure 4.

Global homography showed background blur, duplicated elements (especially on the ground) and projective distortions on the outer borders. Graphcut only displayed some discontinuities in the foreground. The deep learning approach UDIS [34] also displayed some discontinuities and important projective distortions. The mesh-based method REW [25] showed a perfect stitching result (see the definition in Table 2). The non-mesh-based method Perazzi [27] presented a perfect alignment (see the definition in Table 2) with some projective distortions and also introduced new distortions in the overlap area.

In this setup, non-deep learning state-of-the-art methods (REW and Perazzi) outperformed the older techniques (Global homography and Graphcut). The mesh-based technique REW slightly outperformed the non-mesh-based Perazzi method, especially in terms of the distortions. The deep learning approach UDIS, despite providing a much better result than the Global homography baseline, was not as efficient as REW and Perazzi in terms of alignment and distortions.

### 5.2. Experiment 2: Laparoscopic Scenarios

**Figure 5:**
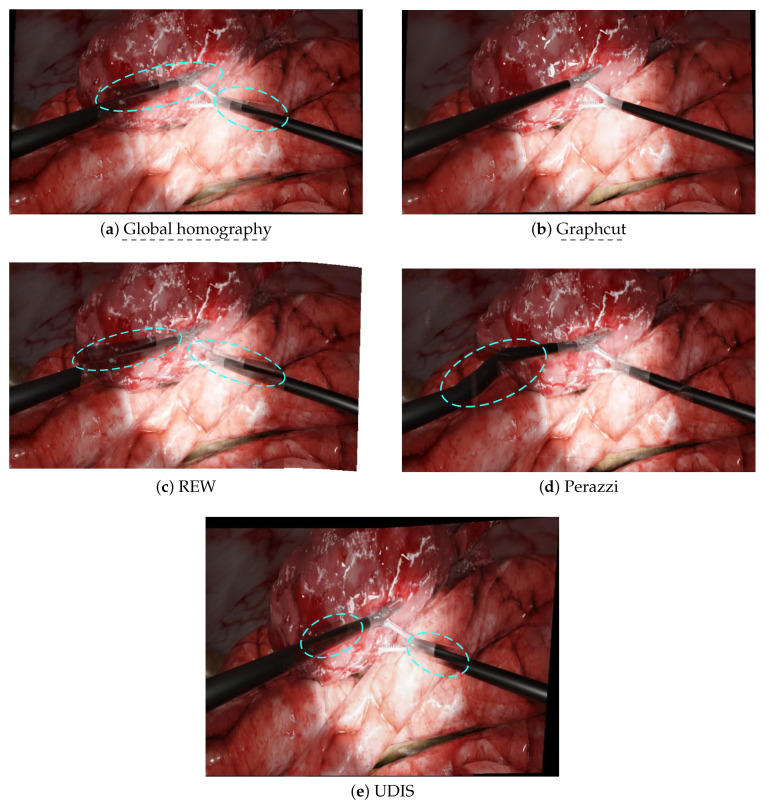
The resulting panoramas in a laparoscopic scenario. Blue dashed circles point to the main issues. (**a**) Global homography: background blur and duplicated forceps. (**b**) Graphcut: perfect stitching. (**c**) REW: no background blur, but duplicated elements (left- and right-hand forceps). (**d**) Perazzi: no duplicated elements, but significant distortions on the left-hand tool. (**e**) UDIS: no background blur, but duplicated elements (left- and right-hand forceps).

In the second set of experiments, we analysed the behaviour of the same algorithms on laparoscopic images generated from our simulated environment. The results are displayed in Figure 5, with input images Figure 3c,d for the left and right images, respectively. Global homography displayed the same problems as in the non-laparoscopic situation. Graphcut performed a perfect stitching. REW, Perazzi and UDIS removed background blur compared to the baseline global homography. Nonetheless, REW and UDIS still displayed duplicated tools and Perazzi introduced significant distortions. In the laparoscopic scenario, Graphcut used in the multi-camera prototype [10] performed better than the state-of-the-art algorithms.

**Figure 6:**
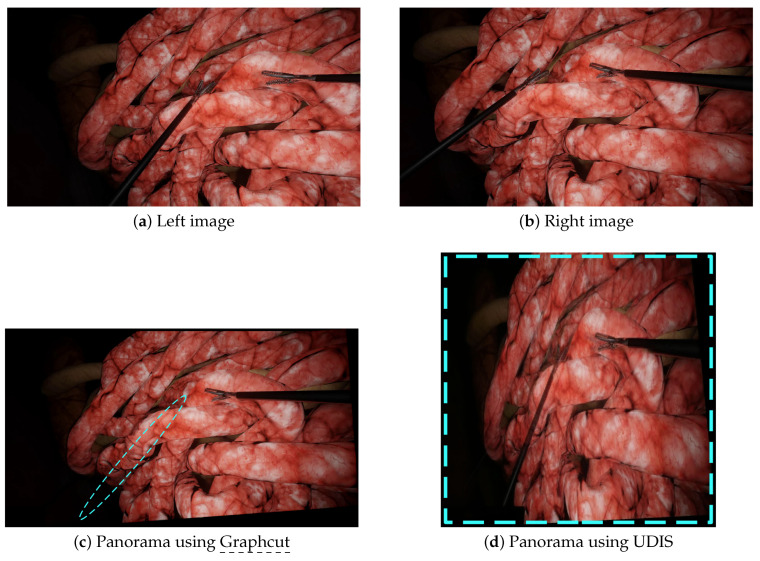
A scenario with more parallax: the left (**a**) and right (**b**) images were stitched together by Graphcut and UDIS. (**c**) With Graphcut, only one tool was present in the final panorama while there were two tools in the input images. (**d**) With UDIS, the two tools appeared in the resulting panorama but with huge projective distortions. The blue dashed lines show the major issues (missing tools and distortions).

**Figure 7:**
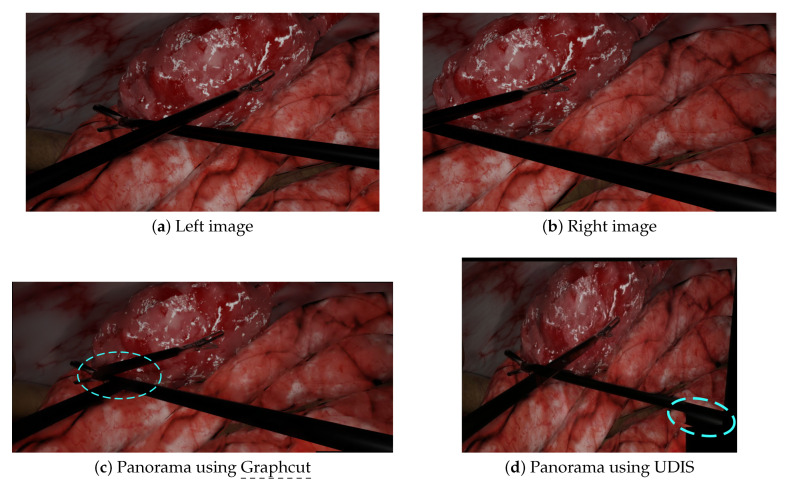
A complex scenario with crossed tools: the left (**a**) and right (**b**) images were stitched together by Graphcut and UDIS. (**c**) With Graphcut, discontinuities appeared when the tools intersected in the input images. (**d**) With UDIS, a duplicated pair of forceps appeared in the bottom right corner.

We performed additional experiments on more complex scenarios with Graphcut and UDIS only, since (a) Graphcut had the best performance in the initial experiments and, (b) to our knowledge, neural networks methods, such as UDIS, have not yet been tested in laparoscopy. The first scenario displayed in Figure 3e,f contains more parallax, while the second in Figure 3g,h contains intersecting tools. In the first scenario, Graphcut, despite a visually pleasant result, generated the panorama with a missing instrument shown in Figure 6c. UDIS, on the other hand, displayed the panorama with the two tools shown in Figure 6d, but with strong projective distortions. In the second scenario, Graphcut clearly introduced strong visible discontinuities, where some tools were just separated into two, as shown in Figure 7c. UDIS provided a better result than Graphcut, as shown in Figure 7d, but still displayed a ghost pair of forceps in the bottom right corner.

The main results are summarised in Table 3.

## 6. Analysis

These experiments showed the limitations of the current techniques used with multi-camera prototypes for laparoscopy.

Global homography, used on prototype [6], was not designed to solve parallax problems since it assumes elements of the scene are coplanar or very far from the cameras (as with mountains in a panoramic photography), which is not true in scenes with very close elements, such as forceps. Thus, this algorithm is not sufficient for use in laparoscopy.

**Figure 8:**
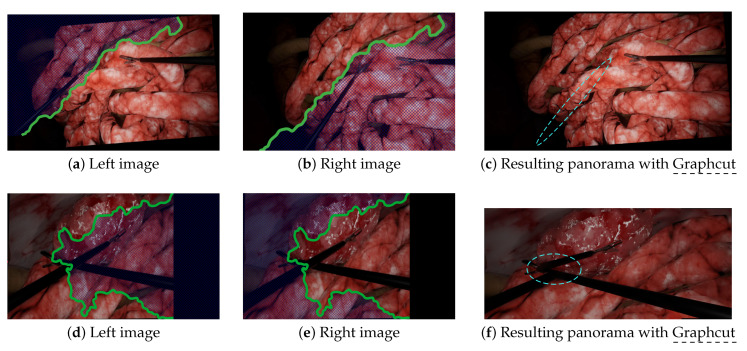
Illustrations of the Graphcut failure to provide reliable image stitching results. Graphcut aligns images by stitching them along a seam (green line). Blue hatching lines represent the areas that disappear during Graphcut alignment. The blue dashed lines show the major issues. (**a**–**c**) In this scenario, the Graphcut algorithm produced a visually satisfying result, to a non-medical expert, but the disappearance of the left forceps would not be acceptable for clinical application (see also Figure 6c). (**d**–**f**) In the scenario with intersecting tools, Graphcut found a seam that passed through the tools, resulting in a significant discontinuity in the final panorama (see also Figure 7c).

Graphcut, despite showing impressive results in some laparoscopic situations, still failed in others due to duplicated/disappearing elements and discontinuities. The disappearing forceps shown in Figure 6c was due to the fact that Graphcut was designed to produce a visually pleasant result and not to ensure that all elements appear in the final panorama. The resulting panorama, despite looking good to a non-medical expert, would not be acceptable for a surgeon. Figure 8a–c illustrates how Graphcut can result in disappearing elements by showing the seam computed by the algorithm. Graphcut’s failure with intersecting tools (cf. Figure 7c) resulted from the fact that the seam inevitably passes over the tools with high parallax resulting in huge discontinuities, as illustrated in Figure 8d–f. This issue was not visible in previous scenarios because the seam passed over the organs with relatively few parallax. Therefore, Graphcut, as used with prototype [10], does not seem reliable enough to be used for multi-camera laparoscopy.

**Figure 9:**
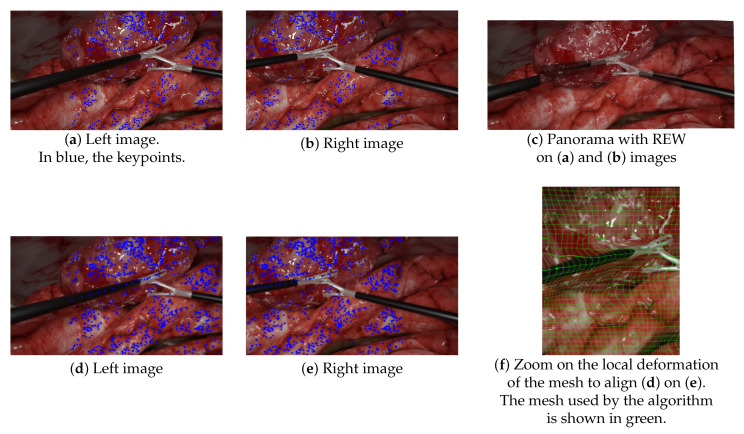
Illustrations of the mesh-based model (REW) failure to provide satisfying results. (**a**,**b**) SURF keypoint detection on the left and right input images. (**c**) The resulting panorama did not align the forceps at all (ghosts), since mesh-based models, such as REW, rely on keypoint matching—keypoints that were missing on the laparoscopic forceps. (**d**–**f**) The same experiments with manually added keypoints along the left tool. REW aligned the tools but induced deformations as illustrated in (**f**).

Surprisingly, these experiments also showed that non-deep learning state-of-the-art techniques, such as REW and Perazzi, do not perform well enough in medical situations. For mesh-based techniques, such as REW, this can be explained mainly by:

- **The lack of keypoints in textureless areas, such as tools**: Figure 9a–c illustrates this lack of keypoints on laparoscopic tools, resulting in the poor alignment of the aforementioned tools. This issue is not specific to our simulated environment, as laparoscopic tools are generally mostly uniform. As previous research that has attempted to propose more textured instruments has never been translated into clinical use, this issue remains a challenge to solve. Kim et al. [10] tried to handle this by replacing keypoint detection with a disparity-based approach that was more robust to textureless areas. However, since their evaluation was performed without any visible instruments, there is no guarantee that it would help to find keypoints on the tools;
- **Mesh-based methods are intrinsically inadequate in situations containing objects in very different planes**. Since mesh-based models compute the continuous deformation of a grid, they expect the parallax issue to be some kind of continuous problem through space too. While being somewhat true in outdoor panoramic photography, this is incorrect in laparoscopy, which contains very thin objects in the foreground. This issue is illustrated in Figure 9d–f. In this experiment, we manually added keypoints along the left tool to force REW to align them. As shown in Figure 9f, it induced a significant local deformation along the tool due to the brutal variation of parallax between the foreground tool and the background. There was not a smooth transition of parallax here.

**Figure 10:**
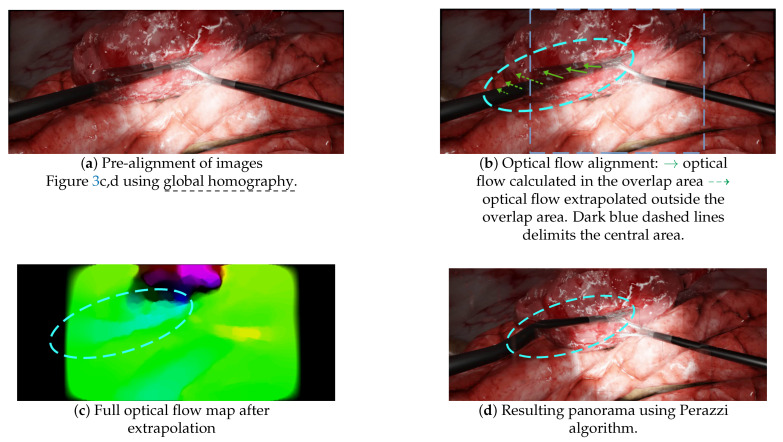
Illustrations of some of the failures of the non-mesh-based method Perazzi. (**a**) The panorama obtained after the pre-alignment step using global homography, with a duplicated left-hand forceps. (**b**) A schematic of the optical flow, computed in the overlap area and extrapolated to outer areas. (**c**) The full optical flow map after “weighted warp” extrapolation. (**d**) The resulting panorama after applying the optical flow map of (**c**) to the pre-aligned images (**a**).

On the other hand, the non-mesh-based technique Perazzi did not suffer from the lack of keypoints, since it mainly relies on optical flow to do the alignment. However, it still contained significant distortions in laparoscopic situations because:

- A pre-alignment is performed using global homography, which introduces projective distortions, as illustrated in Figure 4d;
- Optical flow is then computed in the overlap area and extrapolated to outer areas, as illustrated in Figure 10b,c. This extrapolation, also called “weighted warp extrapolation”, was designed by Perazzi to smoothly join the overlap and non-overlap areas in the panorama. However, this extrapolation is performed uniformly in all directions, ignoring the structures in the image. In the laparoscopic situations, it bent the tools to join them, without considering the expected straightness of forceps, as illustrated in Figure 10d.

UDIS provided better results than any other method in the complex scenarios with crossed tools and high parallax, whilst being unable to compete with REW and Perazzi in the easiest scenario of the temple images. Despite these better results in complex scenarios (see Figure 6d and Figure 7d), UDIS still generated significant distortions and/or duplicated elements:

- The distortions: UDIS relies on a two-step pipeline, with the first step of homography estimation and the second step of refinement. This first homography estimation was not designed to minimise projective distortions and the following step of refinement cannot correct the resulting distortions;
- The duplicated elements: since deep learning approaches are data-driven approaches, it may be more appropriate to train the model with more adapted data, i.e., using thousands of laparoscopic data from our simulated environment. It would, however, require more varied scene backgrounds than those currently available in the environment.

This analysis, besides showing the performance and flaws of each algorithm, also demonstrated the importance of validating multi-camera prototypes in a wide range of laparoscopic scenarios.

## 7. Discussion

As stated in Section 1, our first goal was to provide a simulated environment that was realistic enough to easily evaluate multi-camera prototypes. Ref. [38] also introduced a Blender module for the generation of simulated data for surgery. However, its main goal was to generate various types of data, e.g., depth maps, segmentation masks and optical flow masks in a mono or stereo endoscopic contexts, which are not helpful for the evaluation of image stitching algorithms on multi-camera prototypes. It also offered the possibility to create a 3D scene using an RGB-D recording (which restricts its usage to ex vivo or phantom setups), and some 3D scenes were generated but were not publicly accessible. This motivated us to develop our own simulated environment that would be realistic enough to easily evaluate multi-camera prototypes. Concerning the level of realism of our dataset, we took care to incorporate realistic tools (in terms of textures, shape and sizes) and organ-like textures with dimensions that were coherent with the literature, and we simulated illumination sources adapted to laparoscopic setups (i.e., a spotlight source coming from the tip of the endoscope). While we took care to develop a realistic environment, we cannot provide a quantitative metric proving its degree of realism. However, compared to the environments used by researchers who evaluate multi-camera prototypes (ex-vivo organs, meat or plastic-like organs [6,9,10]), we believe that we were able to progress one step further in terms of realism. Moreover, this environment can be well integrated in the iterative prototyping process. The main direction to further improve this environment would be to make it more realistic in terms of anatomy. We could also add noise effects (e.g., smoke or compression artefacts), which occur during laparoscopy. Nonetheless, our environment was realistic enough to discover and illustrate typical scenarios that were not tested with multi-camera prototypes before. Should the 3D scenes of [38] become available, they could be easily incorporated into our environment to generate more diverse background scenes.

Our second objective was to evaluate the classical and state-of-the-art stitching algorithms within the context of laparoscopy. global homography was not good enough, with blurs, distortions and duplicated forceps. Graphcut, currently used with prototypes such as [10], could make forceps disappear or created significant discontinuities in the resulting panoramas. One way of preventing the disappearance of elements would be to add constraints on the Graphcut method, such that the resulting panoramas keep the same number of tools (e.g., combining it with a tool detection algorithm). The discontinuities with intersecting surgical instruments is another problem that seems intrinsic to this method. State-of-the-art algorithms that performed impressively in non-medical applications displayed poor alignment quality and/or strong distortions in the laparoscopic context. Mesh-based models appear to be intrinsically constrained by their grid. The non-mesh-based method Perazzi appears to be very sensitive to the optical flow accuracy and is not yet reliable enough to be used in a clinical environment. One way of improving this could be to consider the instruments and background as two distinct problems: tool alignment could be performed using tool segmentation in the left and right images, while background alignment could be performed using state-of-the-art algorithms, such as REW. This would, however, require a high-quality segmentation. Another alternative could be to rely on the 3D reconstruction of the scene [7], at the expense of high computational resources. Concerning the promising neural network approaches, such as UDIS, the straightforward approach to improve them for laparoscopy would be to use them with a huge unlabelled dataset from our simulated laparoscopic environment. However, it might not improve the deep learning training to use thousands of images generated from our environment, which does not yet provide enough variety in backgrounds. One possibility would be to use some of the backgrounds from [38], should they become publicly available. This would need to be further investigated, especially to verify that overfitting does not occur as this is a major issue of data-driven techniques.

Finally, this work could be improved by taking the algorithms’ speed into account. We did not focus our work on this question because the algorithms that we benchmarked were not optimised for speed and were coded in various programming languages that would have made a raw speed comparison quite unfair. Our idea was rather to check whether there is an algorithm that performs well enough (qualitatively) in a laparoscopic context, leaving the speed problem as a further issue. Indeed, we do think that there is room for improvement concerning these algorithms: either by using low-level programming languages (C++, even FPGA programming) or by carefully choosing the algorithms’ parameters for a better quality/speed trade-off (e.g., the number of cells for mesh-based models or the optical flow resolution for the Perazzi algorithm). Despite not being real-time, some of the algorithms that we benchmarked were very efficient, even when not being optimised. For instance, Graphcut’s seam estimation required “only” 0.7 s more than global homography for a 620 × 480 image (which could be optimised, for example, by computing the seam on a smaller image resolution: 0.15 s for a 320 × 220 image while leaving the final stitching on the full resolution images). The REW (mesh-based) method had a speed of 2–4 fps (see [25]) and UDIS had a speed of roughly 2 fps (see [34]).

## 8. Conclusions

In this paper, we qualitatively evaluated image stitching algorithms from the non-medical literature, including a promising recent non-supervised deep learning approach, within laparoscopic scenarios. The road to perfect image stitching for laparoscopy seems to be long. Nonetheless, we present here the first assessment of the limits of the current approaches in laparoscopy and the main problems to be solved. Furthermore, we provide a simulated environment to rapidly evaluate multi-camera systems in a quasi-realistic laparoscopic environment. We think that these contributions could help to incentivise the community to work on image stitching for laparoscopy more efficiently.

## Figures and Tables

**Table 1:** The main characteristics of image stitching algorithms from the non-medical literature.

	Basic	Graph Cuts-Based	Mesh-Based Models					Non-Mesh-Based Models	
**Method**	Global Homograpĥy [16]	Graphcut	APAP [21]	PTIS [22]	ANAP [23]	NIS [24]	REW [25]	Perazzi [27]	UDIS [34]
**Alignment over an/along a …**	Overlap region	Seam	Overlap region	Seam	Overlap region	Overlap region	Overlap region	Overlap region	Seam and overlap region
**Features**	SIFT	SIFT	SIFT	SIFT	SIFT	Grid keypoints derived from APAP [21]	SURF	Optical flow	CNN-based
**Method**	Global homography	• Global homography [16] • Graph cuts [19]	• Grid with local homo- graphies • Extrapolation outside of the over- lapping area	Iterative process: • Find a locally coherent homography • Estimate the alignment quality • Refine the alignment using CPW	• Grid with local homo- graphies • Smooth combination of homography and global similarity	Grid with local warp using CPW	• Elastic warp • Grid model to speed up computations	• Pre-alignment with global homography [16] • Optical flow alignment in the overlapping areas • Extrapolation in non-overlapping areas	• Unsupervised global homography estimation • Alignment refi- nement with reconstruction networks, perceptual losses and L1 loss along the seams
**Similarity guidance**	No	No	No	Yes	Yes	Yes	Yes	No	No
**Content-Preserving Warp (CPW)**	No	No	No	Yes	No	Yes	Yes	No	N/A
**Advantages**	• Real time	• Fast • May handle some parallax issues	• Better alignment accu- racy compared to global homography [16] • Solves medium parallax issues	• Combines advantages of seam-based methods and mesh-based methods • May handle important parallax issues	• Fewer projective distor- tions than APAP [21]	• Distortions are globally minimised • Better estimation of the global similarity trans- form compared to ANAP [23] • More natural-looking	• Fast • Minimised distortions	• Adapted to video • Does not rely on keypoints only	• Completely un- supervised • Limited GPU memory requirement • 2 fps
**Drawbacks**	• Not robust to parallax at all (blur, projective distor- tions, duplicated elements)	• Fails on some large parallax issues (discontinuity, projective distortions)	• Projective distortions in non-overlapping areas • Does not handle important parallax issues	• Randomness in the search for optimal homography	• Local distortions when the number of images increases • Non-robust estimation of the global similarity trans- formation (unnatural rotation or scaling)	• Computationally expen- sive	• Fails on important parallax issues	• Bottleneck of optical flow computation	• Do not address the projective distortions.

**Table 2:**
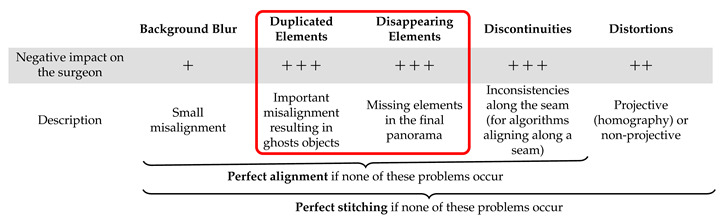
The main problems of image stitching and their potential impacts on surgeons. The most important obstacles to clinical use are highlighted in the red box.

**Table 3:**
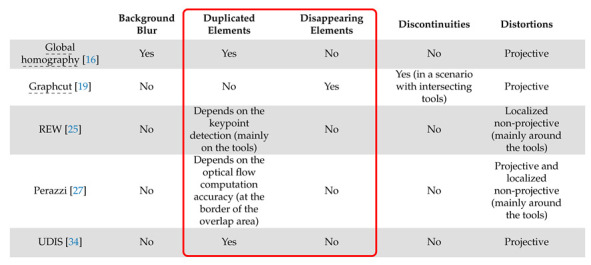
The main limitations of the image stitching algorithms within a laparoscopic context. The most important obstacles to clinical use are highlighted in the red box. Dash-underlined methods are those already employed with multi-camera prototypes.

## Data Availability

The simulated environment and some of the generated images and videos are available at https://gricad-gitlab.univ-grenoble-alpes.fr/guys/laparo_simulated_environment (accessed on 6 January 2022).

## Supplementary Materials

The following are available online at https://www.mdpi.com/article/10.3390/jimaging8030052/s1, Figure S1: Examples of multi-view images rendered in the simulated environment, Figure S2: Additional resulting panoramas on the temple scenario, Figure S3: Additional resulting panoramas on the laparoscopic scenario, Figure S4: Graph of the number of matching keypoints depending on the inter-camera angle and Figure S5: Resulting panoramas in laparoscopic scenario depending on the inter-camera angle.

## Abbreviations

The following abbreviations were used in this manuscript:

ANR	French National Research Agency
CPW	Content-preserving warp
FoV	Field of view
LED	Light-emitting diode
SIFT	Scale-invariant feature transform
SURF	Speeded-up robust features
