# Supplementary material for: Qualitative Comparison of Image Stitching Algorithms for Multi-Camera Systems in Laparoscopy
Source: J Imaging. 2022 Feb 23;8(3):52. doi: 10.3390/jimaging8030052 (PMC8951246; doi:10.3390/jimaging8030052)
Supplement: Supplementary file 1 [file jimaging-08-00052-s001.zip › jimaging-1568735-supplementary.pdf]

Journal of
Imaging

MDPI

Article

Qualitative Comparison of Image Stitching Algorithms for Multi-Camera Systems in Laparoscopy

Sylvain Guy [1], Jean-Loup Haberbusch [1], Emmanuel Promayon [1], Stéphane Mancini [2] and Sandrine Voros [1,3,*]

1 University Grenoble Alpes, CNRS, UMR 5525, VetAgro Sup, Grenoble INP, TIMC, 38000 Grenoble, France; sylvain.guy@univ-grenoble-alpes.fr (S.G.); jean-loup.haberbusch@univ-grenoble-alpes.fr (J.-L.H.); emmanuel.promayon@univ-grenoble-alpes.fr (E.P.)
2 University Grenoble Alpes, TIMA, 38031 Grenoble, France; stephane.mancini@univ-grenoble-alpes.fr
3 University Grenoble Alpes, CNRS, UMR 5525, VetAgro Sup, Grenoble INP, INSERM, TIMC, 38000 Grenoble, France
* Correspondence: sandrine.voros@univ-grenoble-alpes.fr

check for updates

Citation: Guy, S.; Haberbusch, J.-L.; Promayon, E.; Mancini, S.; Voros, S. Qualitative Comparison of Image Stitching Algorithms for Multi-Camera Systems in Laparoscopy. *J. Imaging* **2022**, *8*, 52. https://doi.org/jimaging8030052

Academic Editors: Terry Peters and Elvis C.S. Chen

Received: 7 January 2022
Accepted: 10 February 2022
Published: 23 February 2022

Publisher's Note: MDPI stays neutral with regard to jurisdictional claims in published maps and institutional affiliations.

Copyright: © 2022 by the authors. Licensee MDPI, Basel, Switzerland. This article is an open access article distributed under the terms and conditions of the Creative Commons Attribution (CC BY) license (https://creativecommons.org/licenses/by/4.0/).

Supplementary Material 1

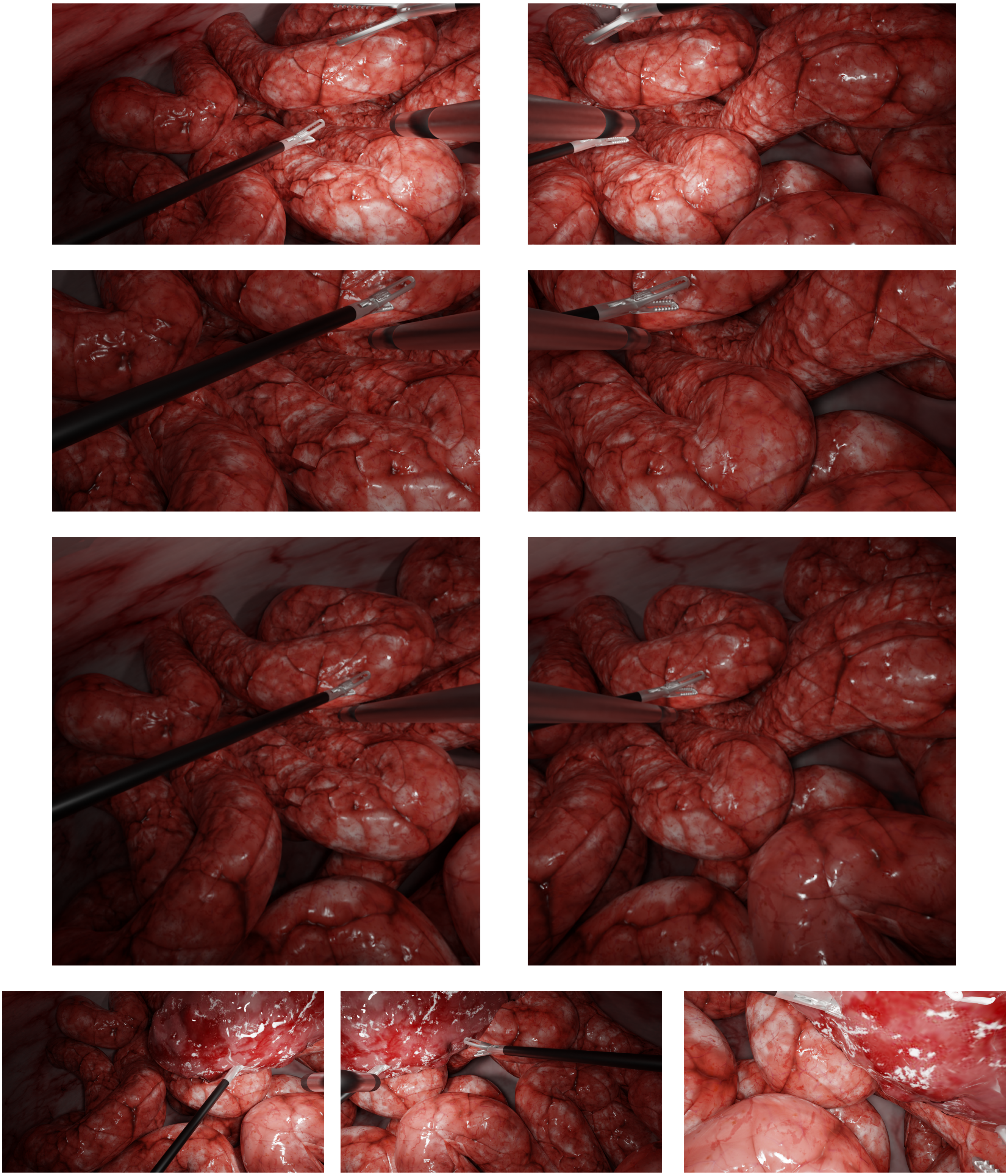

Figure S1. Examples of multi-view images rendered in the simulated environment. Each row is a scenario, left and right columns display respectively the viewpoints of left and right cameras, except the last row that respectively shows the left/right cameras and the endoscope camera. It demonstrates the ability of our environment to use different lighting, image resolutions, cameras orientations.

Supplementary Material 2

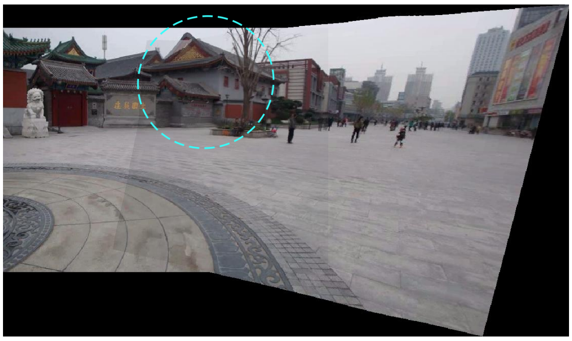

(a) APAP

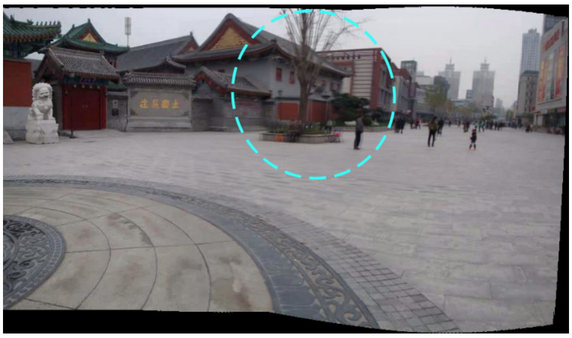

(b) ANAP

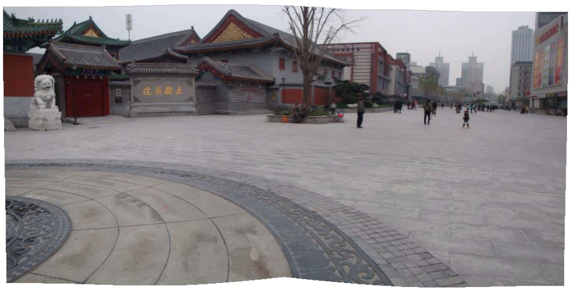

(c) NIS

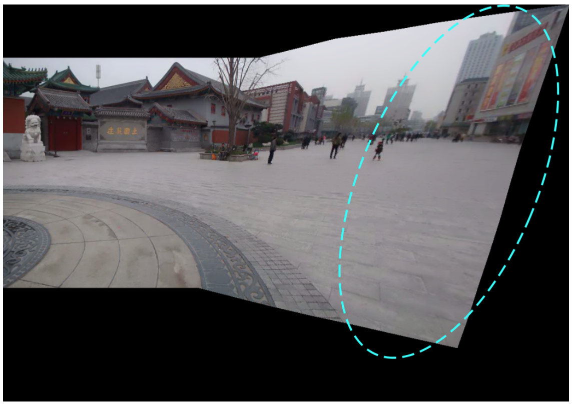

(d) PTIS

Figure S2. Resulting panoramas on the temple scenario. Blue dashed circles point to the main issues. (a) APAP: a bit of background blur, duplicated elements (roof) and projective distortions. (b) ANAP: a bit of background blur and few distortions. (c) NIS: perfect stitching result. (d) PTIS: perfect alignment but important projective distortions.

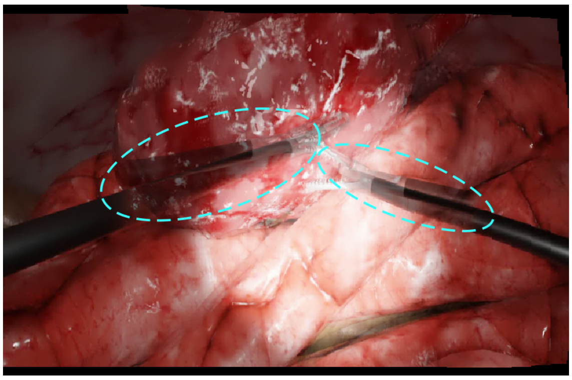

(a) APAP

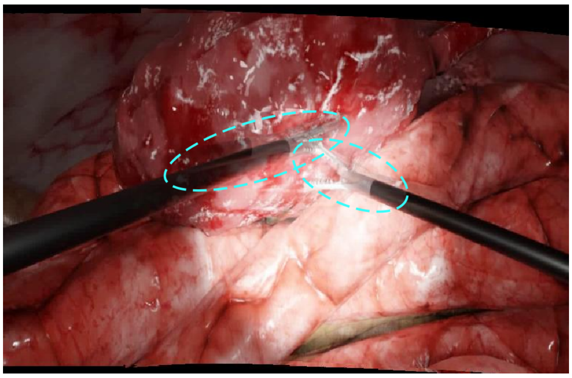

(b) ANAP

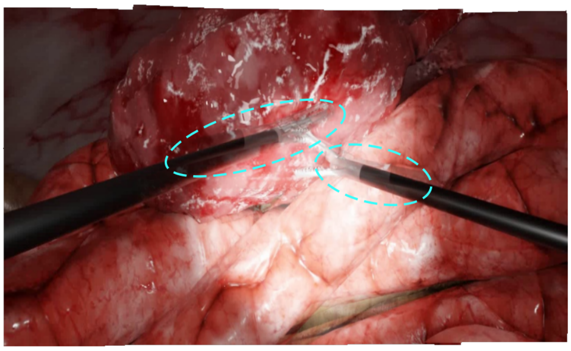

(c) NIS

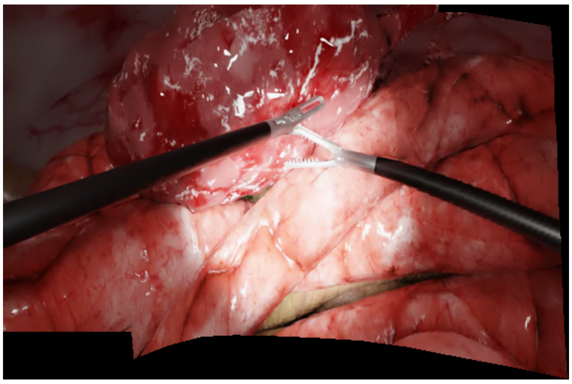

(d) PTIS

Figure S3. Resulting panoramas on a laparoscopic scenario. Blue dashed circles point to the main issues. (a) APAP: duplicated elements (forceps) and background blur. (b) ANAP: duplicated elements (forceps) and background blur. (c) NIS: duplicated elements (forceps) and background blur. (d) PTIS: perfect alignment but a few distortions.

Supplementary Material 3

In this experiment we evaluated how the camera overlap impacts the quality of image stitching results. This experiment is based on the prototype model of [6], with a fixed inter-cameras distance of 4.5cm. Since this prototype enables a varying orientation of cameras (using dedicated screws), we varied the relative orientation of cameras, i.e., the angle between the axes of the 2 cameras or inter-camera angle, in a range from 0 degree (parallel cameras) to 60 degrees. For each of these new orientations, the resulting panorama using Global homography, Graphcut and REW are respectively displayed on Figures S5. This experiment demonstrates that after 24 degrees, the quality of stitching result considerably decreases. This can be put in relation with the graph on Figure S4 that displays the number of matching keypoints between left and right images. This number falls rapidly after 24 degrees too.

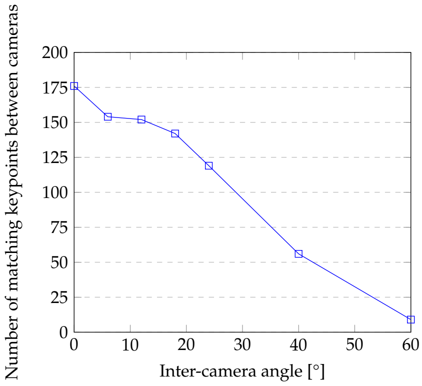

Figure S4. Graph representing the number of matching keypoints between left and right images in function of the inter-camera angle for a fixed inter-cameras distance of 4.5cm.

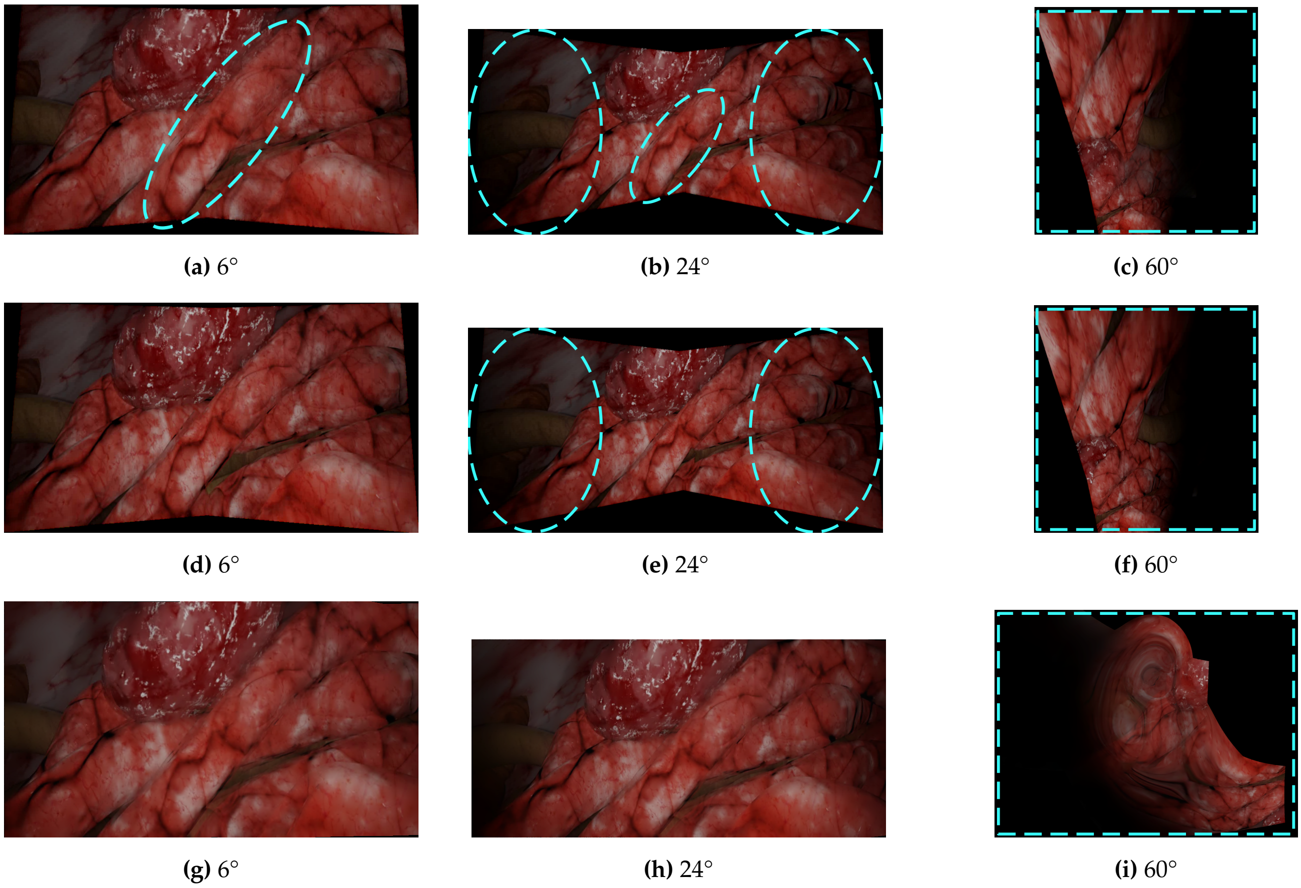

Figure S5. Resulting panorama of Global homography (1st row), Graphcut (2nd row), and REW (3rd row) for increasing inter-cameras angles (6°to 60°). In blue dashed line, the main issues in the panoramas.
